# Antioxidant Activity, Molecular Docking, Quantum Studies and *In Vivo* Antinociceptive Activity of Sulfonamides Derived From Carvacrol

**DOI:** 10.3389/fphar.2021.788850

**Published:** 2021-11-23

**Authors:** Aldo S. de Oliveira, Luana C. Llanes, Ricardo J. Nunes, Catharina Nucci-Martins, Anacleto S. de Souza, David L. Palomino-Salcedo, María J. Dávila-Rodríguez, Leonardo L. G. Ferreira, Adair R. S. Santos, Adriano D. Andricopulo

**Affiliations:** ^1^ Department of Exact Sciences and Education, Federal University of Santa Catarina-UFSC, Blumenau, Brazil; ^2^ Laboratory of Medicinal and Computational Chemistry, Institute of Physics of São Carlos, University of São Paulo-USP, São Carlos, Brazil; ^3^ Department of Chemistry and Biochemistry, University of California, Santa Barbara, Santa Barbara, CA, United States; ^4^ Departament of Chemistry, Federal University of Santa Catarina-UFSC, Florianópolis, Brazil; ^5^ Department of Physiological Sciences, Center of Biological Sciences, Federal University of Santa Catarina-UFSC, Florianópolis, Brazil; ^6^ Department of Structural and Functional Biology, Institute of Biology, University of Campinas-UNICAMP, Campinas, Brazil; ^7^ Department of Chemistry, Federal University of São Carlos-UFSCar, São Carlos, Brazil

**Keywords:** sulfonamides, pain, carvacrol, molecular modeling, antioxidant

## Abstract

The synthesis and antioxidant, antinociceptive and antiedematogenic activities of sulfonamides derived from carvacrol—a druglike natural product—are reported. The compounds showed promising antioxidant activity, and sulfonamide derived from morpholine (**S1**) demonstrated excellent antinociceptive and antiedematogenic activities, with no sedation or motor impairment. The mechanism that underlies the carvacrol and derived sulfonamides’ relieving effects on pain has not yet been fully elucidated, however, this study shows that the antinociceptive activity can be partially mediated by the antagonism of glutamatergic signaling. Compound **S1** presented promising efficacy and was predicted to have an appropriate medicinal chemistry profile. Thus, derivative **S1** is an interesting starting point for the design of new leads for the treatment of pain and associated inflammation and prooxidative conditions.

## Introduction

Pain is a major sorrowful condition that affects children, adolescents (Guindon et al., 2007; Schmidt et al., 2010) and adults (Loeser and Treede, 2008) in several pathologies, including cancer (Ling et al., 2012). Pain can impair daily activities, diminish life quality, and cause significant psychological conditions (Rowlingson, 2000).

Pain is a clinically meaningful sign for the detection and evaluation of many diseases. Its perception is complex, involving two distinct components, an emotional and a physiological or sensorial component, called nociception (Tominaga et al., 2003). Animal models used for the evaluation of antinociceptive activity involve several nociceptive responses generated by chemical, mechanical or thermal stimuli (Silva et al., 2013).

Despite advances in the pharmacokinetics and pharmacodynamics of analgesic agents, their high toxicity is a determinant of conflicting clinical results due to the need for drug associations and interactions, especially in chronic pain due to its bioplasticity, and association with clinical conditions of anxiety and depression that reduce the quality of life of patient.

Sound evidence indicates that amino acids, mainly glutamate, found in C and Aδ fibers, play a fundamental role in the transmission of pain, as they provoke post-synaptic depolarization and the propagation of nociceptive information (Verri et al., 2006). Besides, abnormal excitability caused by inflammation or injury usually results from increased expression or activation of receptors, which may be stimulated by glutamate, favoring the maintenance of the painful stimulus (Rao, 2009; Salvemini et al., 2011). Therefore, substances capable of causing selective changes in glutamatergic signaling may give rise to new analgesic and anti-inflammatory agents.

Upon inflammatory reactions, pro-inflammatory chemical messengers stimulate resident cells, recruit nociceptors and cells, and drive pain conduction (Manchope et al., 2016). Furthermore, augmented oxidative stress upon inflammation promotes nociception. For example, Reactive Nitrogen Species (RNS) and Reactive Oxygen Species (ROS) in a direct and indirect manner promote sensitization and activation of nociceptors (Maioli et al., 2015). The unbalance between oxidative and antioxidative agents in inflammatory reactions promotes oxidative stress (Biswas, 2016). Even though many analgesic agents can be used for the therapy of pain, research on novel drug candidates is needed considering that the current analgesics cause a broad diversity of adverse effects (Burgess and Williams, 2010).

Natural product structural motifs have been an invaluable source of new chemical matter for drug design and medicinal chemistry (Rodrigues et al., 2016). Recently, natural product research in the industry has decreased because of compatibility problems between natural-product extract collections and high-throughput screening platforms (Koehn and Carter, 2005). In this scenario, the monoterpene phenol 2-methyl-5-isopropyl-phenol, known as carvacrol, is a simple molecule with no stereogenic centers, with druglike properties and whose derivatives can be used for structure-activity relationship (SAR) studies. Along with the anti-inflammatory activity of carvacrol (Arigesavan and Sudhandiran, 2015), researchers have been interested in studying the analgesic action of this monoterpene.

**FIGURE 1:**
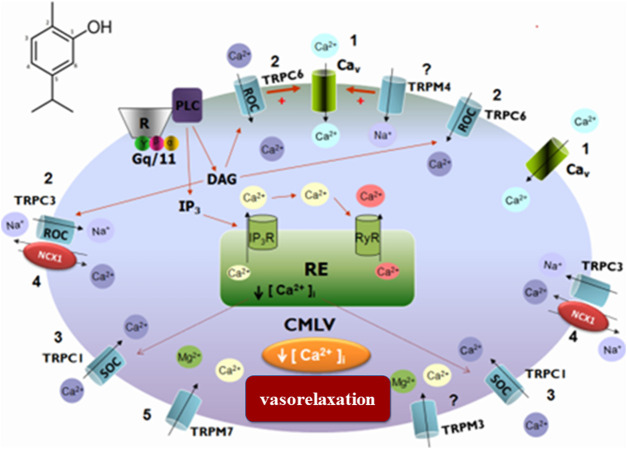
Schematic representation of the probable signaling pathway of the vasorelaxant effect induced by carvacrol. 1) Blockage of calcium influx through the Cav; 2) Blockade of calcium influx through ROC and/or TRPC6; 3) Blockade of calcium influx through SOC and/or TRPC1; 4) Action on NCX1 by activation of TRPC3; 5) Inhibition of TRPM7.

Calcium and potassium channels are also directly related to the transmission of painful impulses since they are central for the release of neurotransmitters from nociceptor terminals. In this sense, studies demonstrate that carvacrol promotes a vasorelaxant response in upper mesenteric artery rings in rats, potentially because it inhibits the influx of calcium ions mediated by voltage-sensitive calcium channels (Cav), as well as the receptor-operated channel (ROC) (Pires et al., 2015). Stock-actuated calcium channels (SOC) seem to be associated with classical TRP receptors (C6, C1, and TRPC) and also with melastatin TRP receptor channel inhibition (TRPM7) (Figure 1). The observed vasorelaxant activity may be involved in the hypotensive response detected in *in vivo* studies (Dantas et al., 2015).

Melo et al. (2010) demonstrated that doses of 12.5, 25, and 50 mg/kg of carvacrol, administered orally, have an anxiolytic effect and do not alter the locomotor activity of the animals. In a previous study, we demonstrated that some synthetic sulfonamides derived from carvacrol at a dose of 30 mg/kg, intraperitoneal (ip), are able to reduce streptozotocin-induced Alzheimer’s disease deficits, in addition to producing anxiolytic and antioxidant effects, without affecting locomotor activity of animals (de Souza et al., 2020). Also, it was confirmed that carvacrol, administered orally, at single doses of 50 and 100 mg/kg, produces significant inhibition of nociception caused by chemical (formalin and acetic acid) and thermal stimulations (hot-plate test) (Cavalcante Melo et al., 2012). Furthermore, part of the mechanism by which carvacrol exerts its effects was demonstrated by Zotti et al. (2013). The authors found that carvacrol administered orally for seven consecutive days (12.5 mg/kg) was able to increase dopamine and serotonin levels in the prefrontal cortex and hippocampus. Following these findings, it has been demonstrated that carvacrol promotes antinociceptive effects by a mechanism that is independent on the activation of the opioid machinery and the L-arginine-nitric oxide (NO) pathway (Cavalcante Melo et al., 2012).

Sulfonamides derived from carvacrol have been investigated recently, for which antibacterial properties (Oliveira et al., 2020) and potential candidates for the development of drugs for the treatment of Alzheimer's disease have been reported (De Souza et al., 2020). As mentioned above and due to the analgesic and anti-inflammatory potential of carvacrol, in this research, the antinociceptive potential of these sulfonamides was investigated. Thus, this investigation is the first report to demonstrate the potential antioxidant activity of sulfonamides derived from carvacrol. Furthermore, this is the first report of sulfonamides derived from carvacrol, rationally designed to the effective control of pain via inhibition of the glutamatergic system. Additionally, molecular docking and quantum investigations were carried out to rationalize the *in vitro* and *in vivo* data.

Despite advances in the pharmacokinetics and pharmacodynamics of analgesic agents, their high toxicity is a determinant of conflicting clinical results due to the need for drug associations and interactions, especially in chronic pain due to its bioplasticity, and association with clinical conditions of anxiety and depression that reduce the quality of life of patient (Berman and Bausell, 2000; Jensen et al., 2001). Therefore, the development of new chemotherapeutic agents for pain treatment, which is the objective of this research, is extremely relevant in the context of public health worldwide.

## Materials and Methods

### Synthesis of Sulfonamides

All the solvents used were analytically pure. The reagents 5-isopropyl-2-methylphenol (carvacrol), chlorosulfonic acid, morpholine, 4-fluoroaniline, pyridin-2-yl methanamine, 2-hydroxyaniline, 2,4-dichloroaniline were obtained from Sigma Aldrich.

**SCHEME 1:**
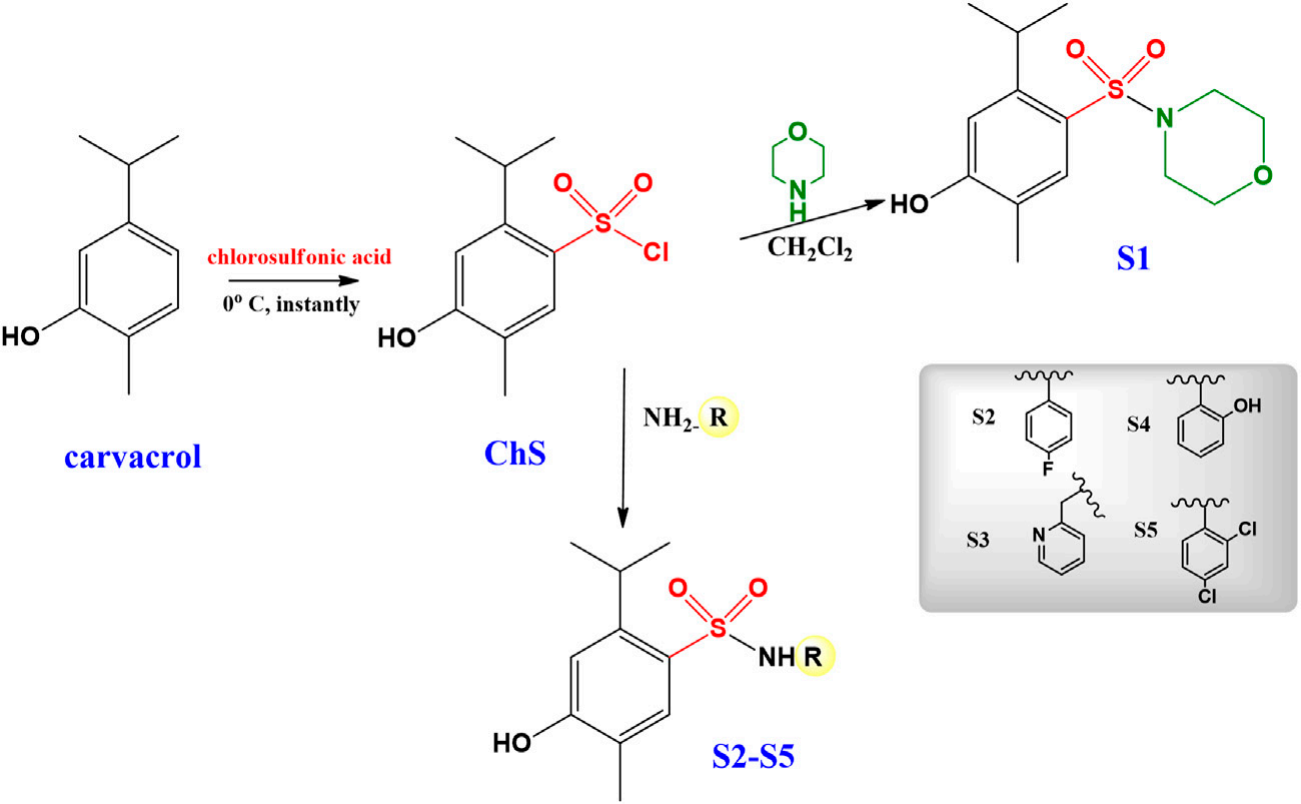
Synthesis of the carvacrol-derived sulfonamides.

The synthesis sulfonamides **S1–S5**, as already described in the literature (de Oliveira et al., 2016) was performed in two steps: firstly, the synthesis of 4-hydroxy-2-isopropyl-5-methylbenzene-1-sulfonyl chloride (ChS) was performed, subsequently, the ChS was used in reactions with different amines (Scheme 1). ChS was obtained from the reaction of carvacrol to six equivalents of chlorosulfonic acid. The sulfonamides obtained in this study were prepared from ChS with two equivalents of amine added slowly. Reactions were followed by thin layer chromatography (TLC). All sulfonamides were purified by acid-base extraction and the compounds were duly characterized by spectroscopic and spectrometric techniques.

### Behavioral Tests

#### Animal Models

Animal care and *in vivo* procedures were carried out according to the ethical guides for the study in conscious animals of experimental pain (Zimmermann, 1983). The experiments were carried out after protocol approval from the Ethics Committee of the Federal University of Santa Catarina—UFSC (protocol PP00745). Male Swiss mice (25–35 g) were obtained from UFSC. Animals were maintained in a 12 h light/12 h dark cycle (lights on at 6:00 a.m.) under a temperature of 22 ± 2°C with water and food *ad libitum*. At least 1 h before the tests, the animals were acclimatized to the laboratory conditions. The tests were executed from 8:00 a.m. to 12:00 a.m. The number of animals and noxious stimulation intensity were kept at the minimum needed to obtain consistent results.

#### Drugs and Reagents

The following substance was used: L-glutamic acid hydrochloride (Sigma–Aldrich, St. Louis, MO, United States). This formulation has a glutamate content of ≥99% measured by HPLC, according to the manufacturer's technical sheet. The carvacrol, used in this work, was obtained commercially in liquid form by Sigma-Aldrich, whose density is 0.976 g/ml at 20°C (lit.), melting point 3–4°C (lit.) with a concentration of 98 %. Glutamate was solubilized in isotonic saline solution (0.9% NaCl), and carvacrol and sulfonamides derived from carvacrol (**S1–S5**, Scheme 1) were dissolved in saline plus Tween 80. Tween 80 did not exceed a 5% final concentration and did not show any activity by itself. Control groups for each delivery route were given isotonic saline with Tween 80 at 5%.

#### Glutamate-Induced Nociception

To demonstrate the possible interplay between the carvacrol derivatives and the glutamatergic system, we evaluated whether the compounds would antagonize the glutamate-induced pain behavior of paw licking and biting. This glutamate-induced model of nociception was reported previously (Beirith et al., 2002; Meotti et al., 2010). A 20 µl glutamate solution (20 µmol/paw, in saline, with pH adjusted to 7.4) was administered intraplantarly (i.pl.) in the ventral face of the right hind paw. After the administration of glutamate, the mice were monitored for 15 min. Nociception was monitored by measuring with a chronometer the amount of time that mice spent licking and biting the injected paw. The mice were given vehicle intragastrically (i.g.) (10 ml/kg) or carvacrol derivatives (0.0003, 0.003, and 0.03 mg/kg) 1 h before glutamate administration.

Additionally, the thickness of the animal paw was measured with a digital micrometer (0–25 mm) before and after the nociceptive response induced by glutamate (i.pl.) to evaluate the paw edema. The difference in thickness (mm) of the hind paw, immediately before and after the test of glutamate, was considered as an index of edema.

#### Evaluation of Locomotor Activity

The open-field test is widely used to assess spontaneous locomotor activity in animals to exclude possible nonspecific effects of a drug on the central nervous system (CNS), causing sedation or motor dysfunction. This is an important measure to check for possible false positives in pain studies, as these parameters can be easily confused with an analgesic effect of the evaluated drug and cause research bias. Thus, to examine the activity of the carvacrol derivatives on spontaneous locomotion, the open-field test was performed as described above (Nucci-Martins et al., 2016; de Souza et al., 2020). The open-field test device was a wooden box (40 × 60 × 50 cm). The floor was split into 12 equal squares, and the number of squares that the animal covered with all paws in a 6 min session was registered. Mice were given the compounds (i.g., 0.0003, 0.003, and 0.03 mg/kg) or vehicle (i.g., 10 ml/kg) 1 h before the test. Healthy mice that were not submitted to painful stimuli were used for the assessment of locomotor activity in the open-field experiment.

### Statistical Analyses

Results are reported as average values ± standard deviation (SD) with the exception of ID_50_ and EC_50_ values, which were calculated from single experiments using nonlinear regression implemented in GraphPad 7.0 (GraphPad software, San Diego, CA, United States). The glutamate test with paw edema measurement and the open-field test showed a normal data distribution in line with the Shapiro–Wilk threshold (*p* = 0.05) and, thus, were submitted to one-way ANOVA analysis and to Dunnett test for multiple analyses. Only *p*-values below 0.05 were taken as significant (*p* < 0.05).

### Antioxidant Assays

#### Scavenging Assay—Nitric Oxide

NO scavenging assay was performed using the method reported by Sens et al. (2018). In this assay, sodium nitroprusside generates NO radicals (NO•) which react with oxygen to generate nitrite ions. The production of the nitrite ions is then determined with the Griess reagent (1% sulfanilamide, 2% H_3_PO_4_ and 0.1% naphthylethylenediamine dihydrochloride). NO scavenging activity was measured by adding 1.5 ml phosphate buffer saline (0.2 M, pH 7.4) and 1 ml sodium nitroprusside (10 mM) to several concentrations of the test compounds (25, 50, 75, and 100 mg ml^−1^) and incubating the reaction mixture for 150 min (25°C). Next, 1 ml of Griess reagent was added to 1 ml of the reaction solution. A wavelength of 546 nm was set to measure absorbance (A), and the results of antioxidant assays were expressed as EC_50_.

#### Scavenging Assay—Hydrogen Peroxide

The H_2_O_2_ scavenging activity showed by the compounds was measured spectrophotometrically using a method reported previously (Sens et al., 2018). A 40 mM H_2_O_2_ solution was made in phosphate buffer (pH 7.4). 25, 50, 75, and 100 mg ml^−1^ test compound solutions in phosphate buffer (3.4 ml) were added to the H_2_O_2_ solution (0.6 ml). Absorbance was monitored at a wavelength of 230 nm. The percentage of H_2_O_2_ scavenging was calculated, and the results were expressed as EC_50_.

### Computational Studies

#### Small-Molecule Modeling and Preparation

All compounds were built in the Avogadro program (Hanwell et al., 2012). The structures of the compounds were optimized at pH 7.4 to simulate the conditions found experimentally. Next, the compounds were minimized with the MMFF94s force field (Halgren, 1996) and the conjugate gradient method.

#### Density Functional Theory

All energy values of the lowest unoccupied molecular orbitals (LUMO) and highest occupied molecular orbitals (HOMO) were computed by the GAMESS (General Atomic and Molecular Electronic Structure System) software (Schmidt et al., 1993). In the calculation of simple energy, the Becke’s three-parameter hybrid functional, the Lee–Yang–Parr correlation (B3LYP) functional (Nageswari et al., 2018) and the 6–31G(d, p) basis set were used in these molecular systems in gas phase, considering the neutral and singlet structures. The computation was run considering the Slater exchange potential correlation and the grid methodology. The Hückel method (Hückel, 1931) generated an initial estimate of molecular orbitals and electronic density. Consequently, the self-consistent field (SCF) convergence was attributed by the restricted Hartree-Fock (RHF) method (Schmidt et al., 1993), which was limited to 30 iteration cycles. LUMO and HOMO potentials were compared with the experimental results of NO (EC_50_^NO^) and peroxide (EC_50_^H2O2^) elimination activities. Finally, HOMO-biological activity (EC_50_^NO^ and EC_50_^H2O2^) linear regression models were developed.

#### Molecular Docking

The PDB (Berman et al., 2002) was searched for structures of *Rattus norvegicus* bound to antagonist corresponding to the UniProt Gene Names Grin1 and Grin2A-D (NMDA receptors; 23 structures found); Gria1-4 (AMPA receptors; 16 structures found); Grik1-5 (Kainate receptors; 20 structures found); Grm1 and Grm5 (mGluR Group I receptors; 1 structure found); Grm2–3 (mGluR Group II receptors; no structures found) and Grm4–8 (mGluR Group III receptors; no structures found). When more than one structure was available, a direct comparison of the binding sites was performed to evaluate their plasticity and select the smallest subset of structures capable of representing it. For each subset, ensemble docking calculations were performed. After identifying the structure of each receptor with a higher affinity for the compounds, docking simulations were performed individually. The structural data of the heme domain of rat neuronal NO synthase bound to 6-(3-fluoro-5-(3-(methylamino)prop-1-yn-1-yl)phenethyl)-4-methylpyridin-2-amine (PDB 6NGJ) was additionally used.

In all docking calculations, performed with GOLD v.5.6.1 and the ChemPLP (Korb et al., 2009) scoring function, the receptors were kept rigid, and the ligands were treated with full flexibility. The receptors were prepared using GOLD, and structural water molecules were not considered. The atoms up to a distance of 8 Å from the crystallographic ligands in both the ensemble and individual docking simulations were considered to define the binding sites. PyMOL v.1.8 (Schrödinger, New York, NY) was used to create the receptor-ligand figures.

#### Molecular Properties and Pharmacokinetics

Molinspiration Chemoinformatics was used for calculating Octanol-Water Partition Coefficient (milogP), number of atoms (natoms), Topological Polar Surface Area (TPSA), molecular weight (MW), hydrogen bond donors (HBD) and hydrogen bond acceptors (HBA), rotatable bonds (NRB), Molecular Volume, and Lipinski RO5 violations.

The SwissADME tool (http://www.swissadme.ch) was employed for the generation of the Bioavailability Radar, and assess lipophilicity, druglikeness, medicinal chemistry and pharmacokinetics parameters.

## Results

The synthetic procedures for the sulfonamides **S1–S5** (Scheme 1), following a recently reported methodology (de Oliveira et al., 2016), were performed in good yields (85–95%).

### Antioxidant Activity

The antioxidant activity of the sulfonamides derived from carvacrol (Table 1) was analyzed by the NO and H_2_O_2_ scavenging activity assays.

**TABLE 1:** Antioxidant activity of sulfonamides derived from carvacrol.

Compound	NO scavenging activity EC_50_ (µM)	H_2_O_2_ scavenging activity EC_50_ (µM)
S1	12.25 ± 0.12	13.13 ± 0.11
S2	18.11 ± 0.14	20.16 ± 0.17
S3	12.14 ± 0.28	13.85 ± 0.33
S4	18.76 ± 0.22	20.28 ± 0.14
S5	12.04 ± 0.11	13.12 ± 0.18
Ascorbic acid	14.72 ± 0.23	16.3 ± 0.26

### Quantum Studies

**FIGURE 2:**
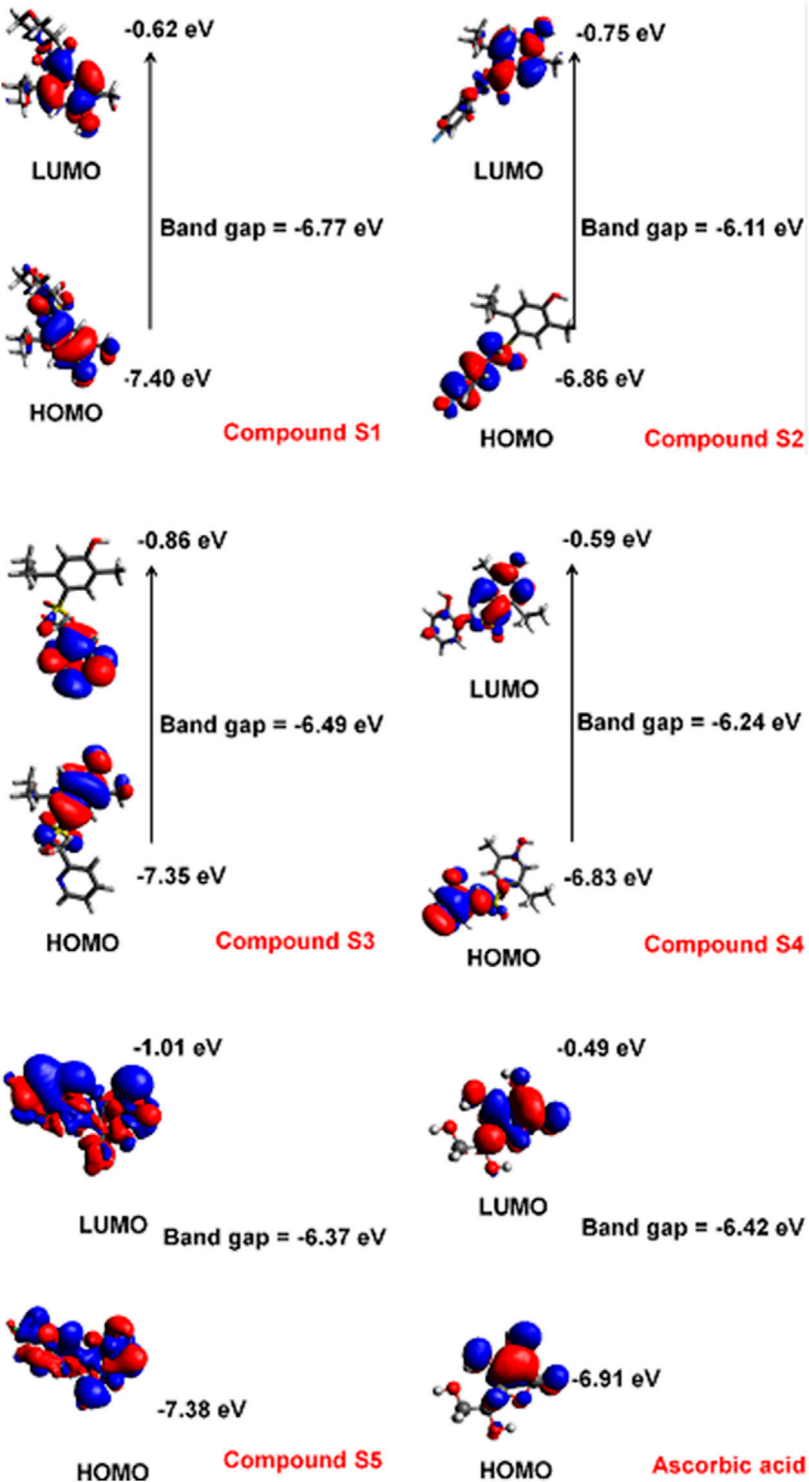
HOMO and LUMO potentials of the carvacrol derivatives estimated by the B3LYP method and 6–31G(d,p) basis set.

The electronic properties were directly correlated with the antioxidant activity of the molecules. The E_HOMO_ and E_LUMO_ indicate the molecule’s ability to donate and receive electron density, respectively. The difference between the two energy levels is termed the band gap and gives an estimate of the reactivity of a molecule. The distance between the HOMO and LUMO energy levels is inversely proportional to the reactivity the compound. The HOMO and LUMO potentials and band gap of the carvacrol derivatives are shown in Figure 2.

**FIGURE 3:**
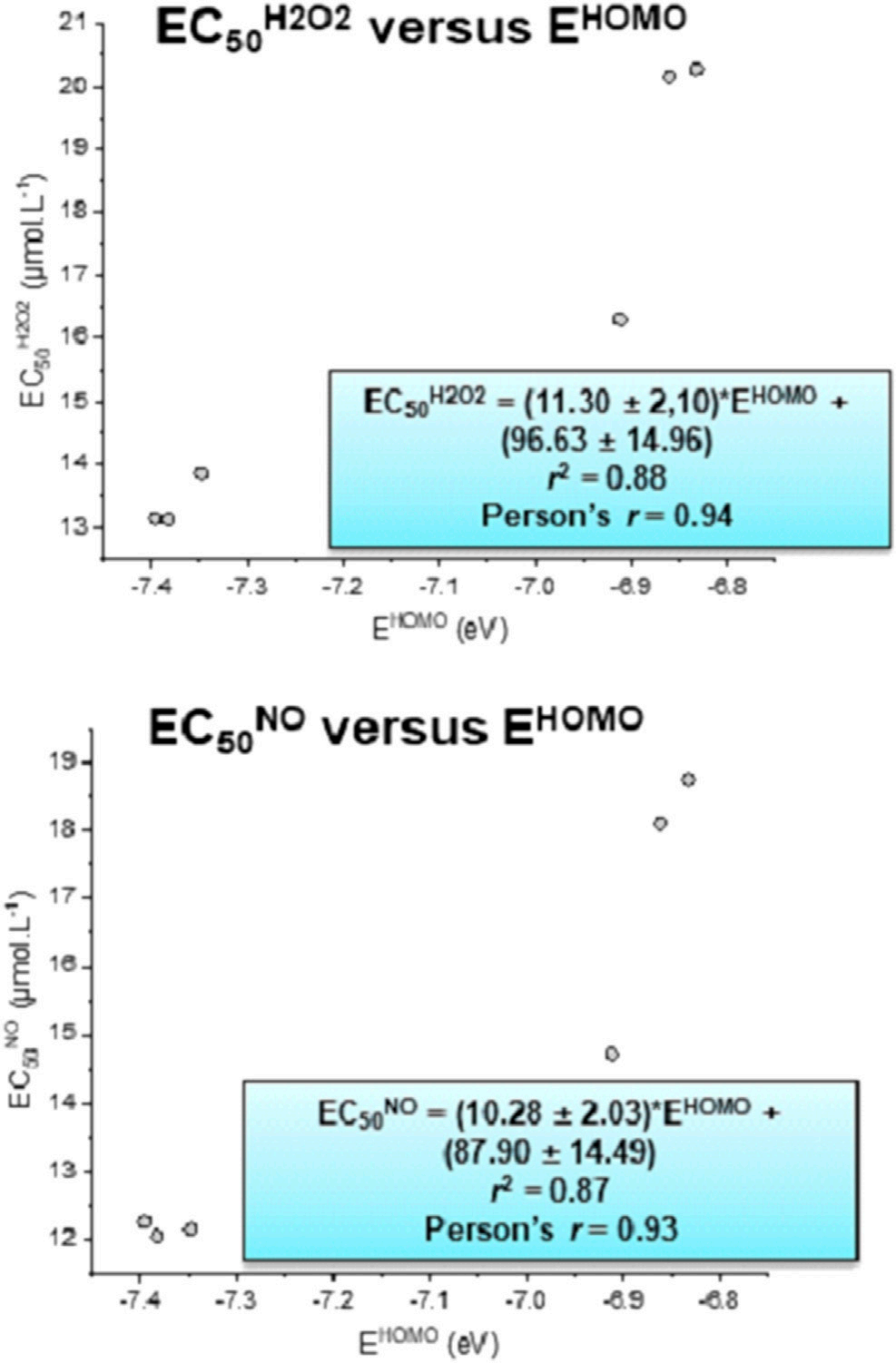
HOMO energy (E^HOMO^) correlated with EC_50_^NO^ and EC_50_^H2O2^.

Figure 3 shows the correlation between HOMO energy and experimental EC_50_^NO^ and EC_50_^H2O2^. The correlation coefficients *r* ^2^ and Person's coefficient (*r*) of the EC_50_^NO^ versus E^HOMO^ were 0.87 and 0.93, respectively. For EC_50_^H2O2^ versus E^HOMO^ *, r* ^2^ and *r* were 0.88 and 0.94, respectively. The angular coefficient values of the equations EC_50_^NO^ = EC_50_^NO^ (E^HOMO^) and EC_50_^H2O2^ = EC_50_^H2O2^ (E^HOMO^) were, respectively, 10.28 ± 2.03 and 11.30 ± 2.10 µmol. (L.eV)^−1^. In addition, the linear coefficients were 87.90 ± 14.49 and 96.63 ± 14.96 µmol. (L.eV)^−1^, respectively. From these equations, the minimal values of E^HOMO^ (i.e., EC_50_^NO^ = EC_50_^H2O2^ = 0) can find the maximal activity. Thus, with the HOMO energy tending to −8.55 eV for both equations, the maximal elimination of NO and H_2_O_2_ is reached for both experiments.

### Antinociceptive Activity

**FIGURE 4:**
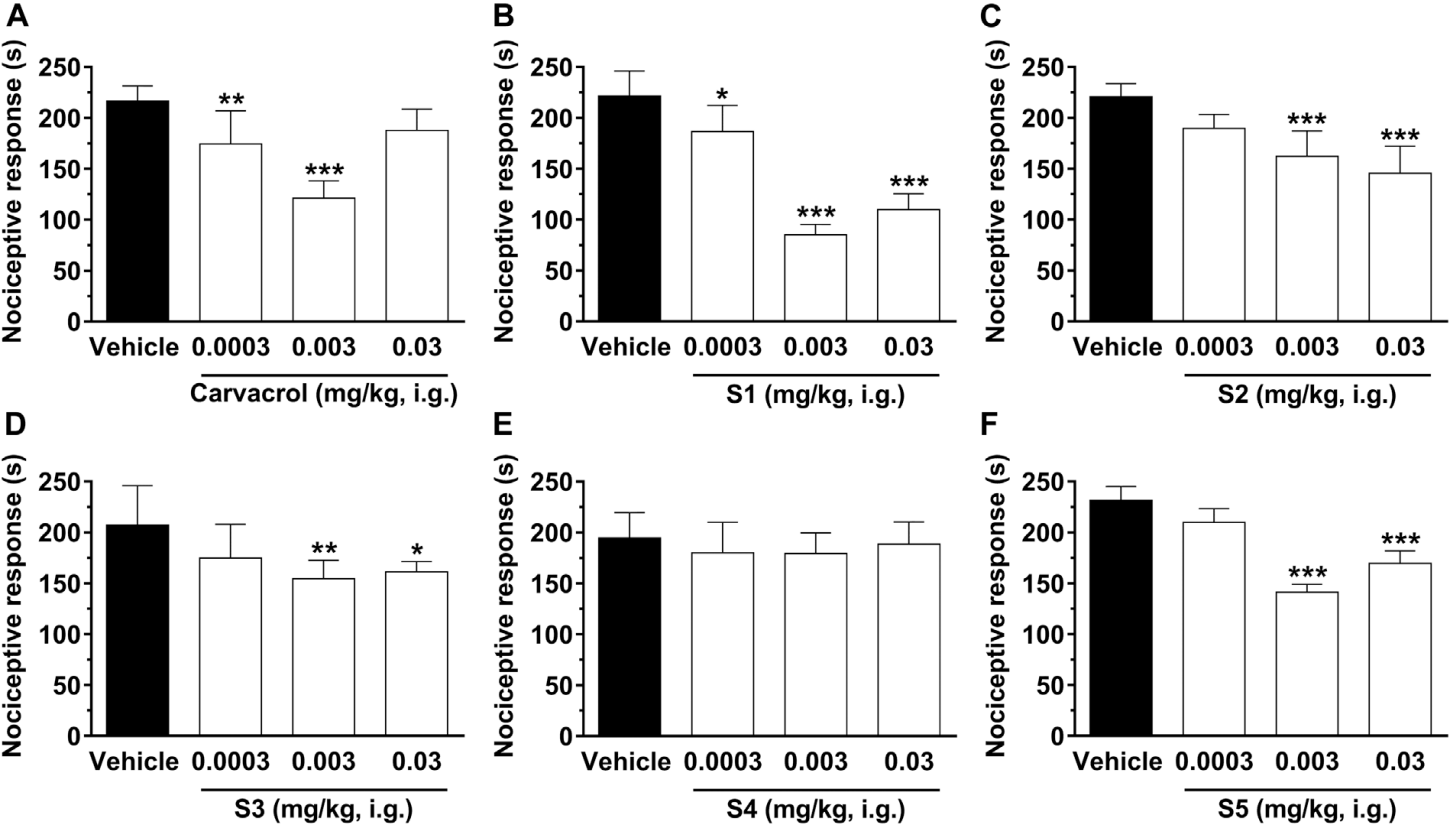
Effect of compounds on nociception induced by glutamate (i.pl.) in mice. The pain behavior, translated by the nociceptive response of licking/biting hind paws induced by glutamate (i.pl.), was evaluated 1 hour after treatment with carvacrol **(A)**, **S1 (B)**, **S2 (C)**, **S3 (D)**, **S4 (E)** and **S5 (F)** at doses ranging from 0.0003, 0.003, and 0.03 mg/kg, i.g., (open bars) or vehicle/control (closed bar). Each bar denotes the average response for 6–8 animals, and the vertical lines represent the SEM (standard error of mean). Asterisks (*) indicate the significance in comparison with the control group animals (*p < 0.05, **p < 0.01, and ***p < 0.001). One-way ANOVA and Dunnett test for multiple comparisons were used to determine the statistical significance.

For a better understanding of the antinociceptive effect of sulfonamides derived from carvacrol (**S1–S5**), we used the model of glutamate-induced (i.pl.) nociception. This method allowed us to investigate the possible interaction of peripheral antinociceptive action of the analyzed compounds with the glutamatergic system. The results are shown in Figure 4.

**FIGURE 5:**
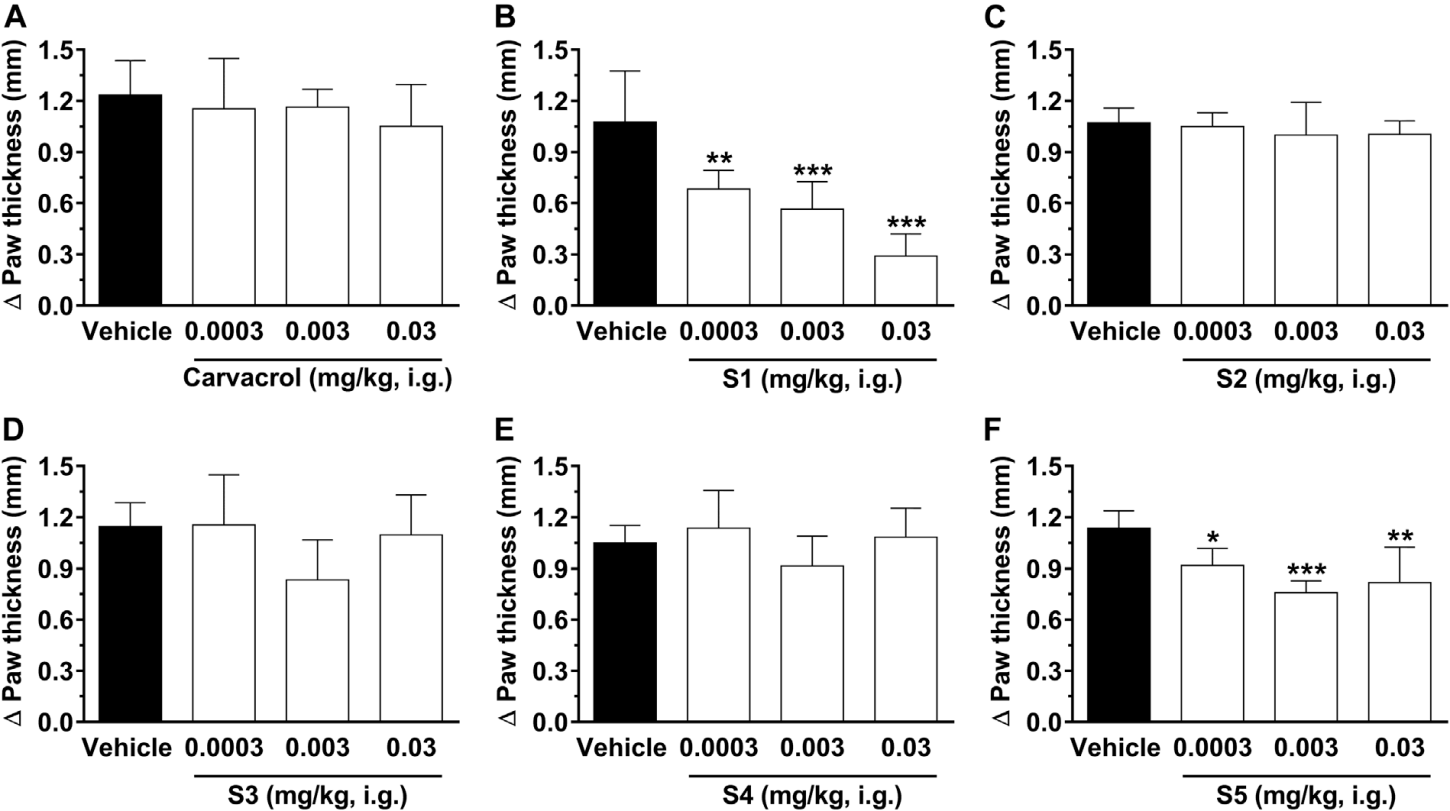
Effect of compounds on paw edema in rats induced by glutamate (i.pl.). The edema was evaluated 1 hour after treatment with carvacrol **(A)**, **S1 (B)**, **S2 (C)**, **S3 (D)**, **S4 (E)** and **S5 (F)** at doses ranging from 0.0003, 0.003, and 0.03 mg/kg, i.g., (open bars) or vehicle/control (closed bar). The animal paw thickness difference was observed before and after the glutamate test. Each bar denotes the average response for 6-8 animals, and the vertical lines represent SD. Asterisks (*) indicate the significance in comparison with the control group animals (*p < 0.05, **p < 0.01, and ***p < 0.001). One-way ANOVA and Dunnett test for multiple comparisons were used to determine the statistical significance.

Figure 5 shows the results of treatment with carvacrol and its derivatives on paw edema induced by glutamate (i.pl.). Our results show that only **S1** and **S5** were able to significantly reduce edema. However, **S1** inhibited edema more effectively and dose-dependently. The percent inhibition values were: 36 ± 10%, 47 ± 6%, and 73 ± 12% for **S1** at 0.0003, 0.003 and 0.03 mg/kg i.g., respectively; 19 ± 9%, 33 ± 6%, and 28 ± 7% for **S5** at 0.0003, 0.003, and 0.03 mg/kg i.g., respectively. The value of ID_50_ for compound **S1** was 0.002 (0.0009–0.005) mg/kg. Furthermore, the calculated values for the ID_50_ antiedematogenic effect of **S1** (0.002 mg/kg) agree with the dose found in the glutamate test, showing homogeneity of the data in this group. Thus, we suggest that **S1** may be an interesting target for the reduction of edema in inflammatory conditions.

**FIGURE 6:**
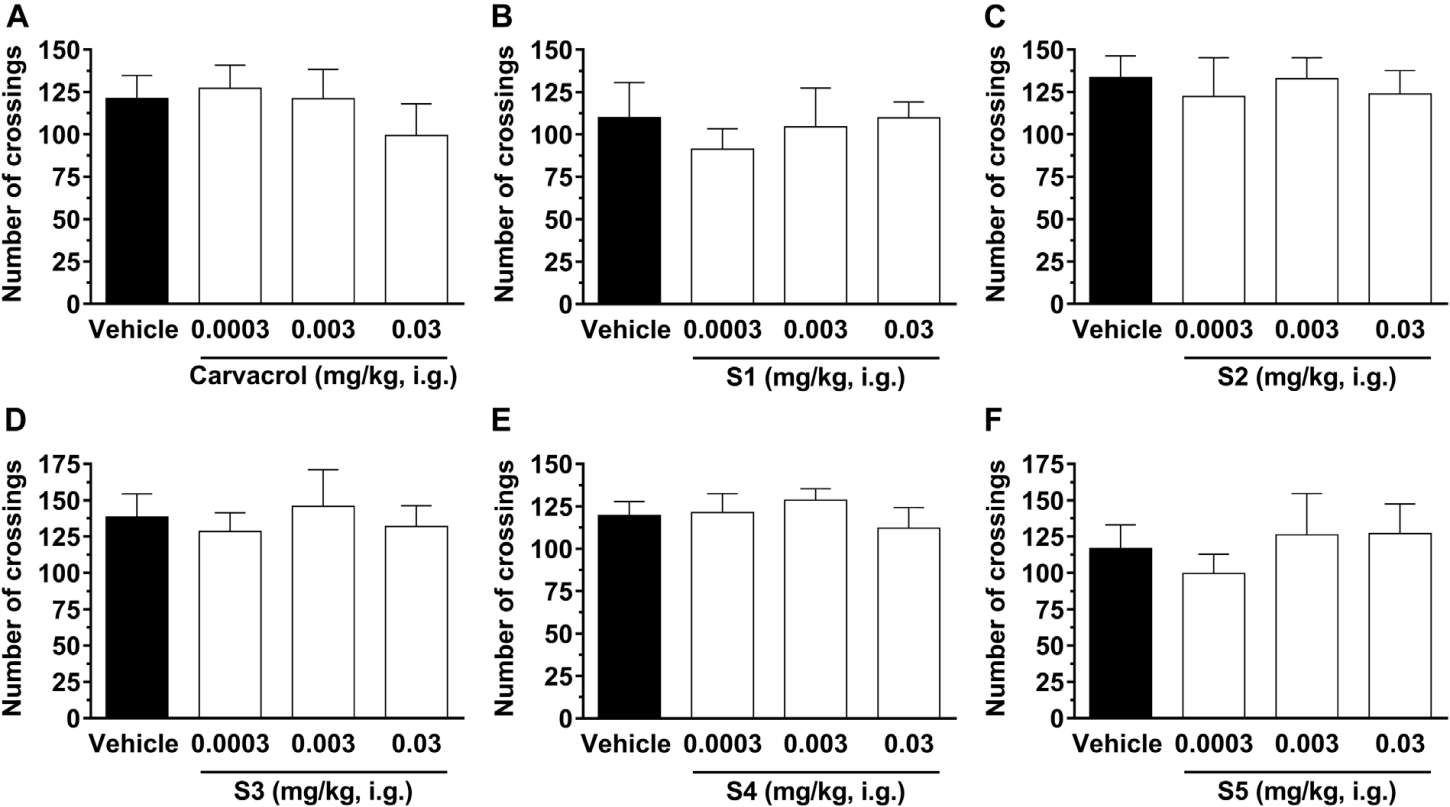
Effect of compounds on the spontaneous locomotion of animals. The crossings were evaluated 1 h after treatment with carvacrol **(A)**, **S1 (B)**, **S2 (C)**, **S3 (D)**, **S4 (E)** and **S5 (F)** at doses ranging from 0.0003, 0.003, and 0.03 mg/kg, i.g., (open bars) or vehicle/control (closed bar). Each bar denotes the average values for 6–8 animals, and the vertical lines represent SD. One-way ANOVA and Dunnett test for multiple comparisons were used to determine the statistical significance.

Figure 6 shows that intragastric administration of carvacrol and compounds **S1**, **S2**, **S3**, **S4**, and **S5** at doses ranging from 0.0003 to 0.03 mg/kg had no effect on the locomotion of animals in comparison with the animals in the control group, suggesting that the compounds do not induce impairment of motor function in the animals. These results exclude the possibility that the antinociceptive action of carvacrol and its derivatives is nonspecifically associated with activity on the peripheral or central levels of locomotion control, such as sedation or motor dysfunction.

### Molecular Docking

As previously shown (Fundytus, 2001), the administration of glutamate receptor (GluR) antagonists has an analgesic effect on peripheral pain. To assess whether the mechanism of action of **S1–S5** is likely to involve these receptors, molecular docking simulations were performed over different GluR structures of *Rattus norvegicus* bound to antagonists (Supplementary Table S1).

**FIGURE 7:**
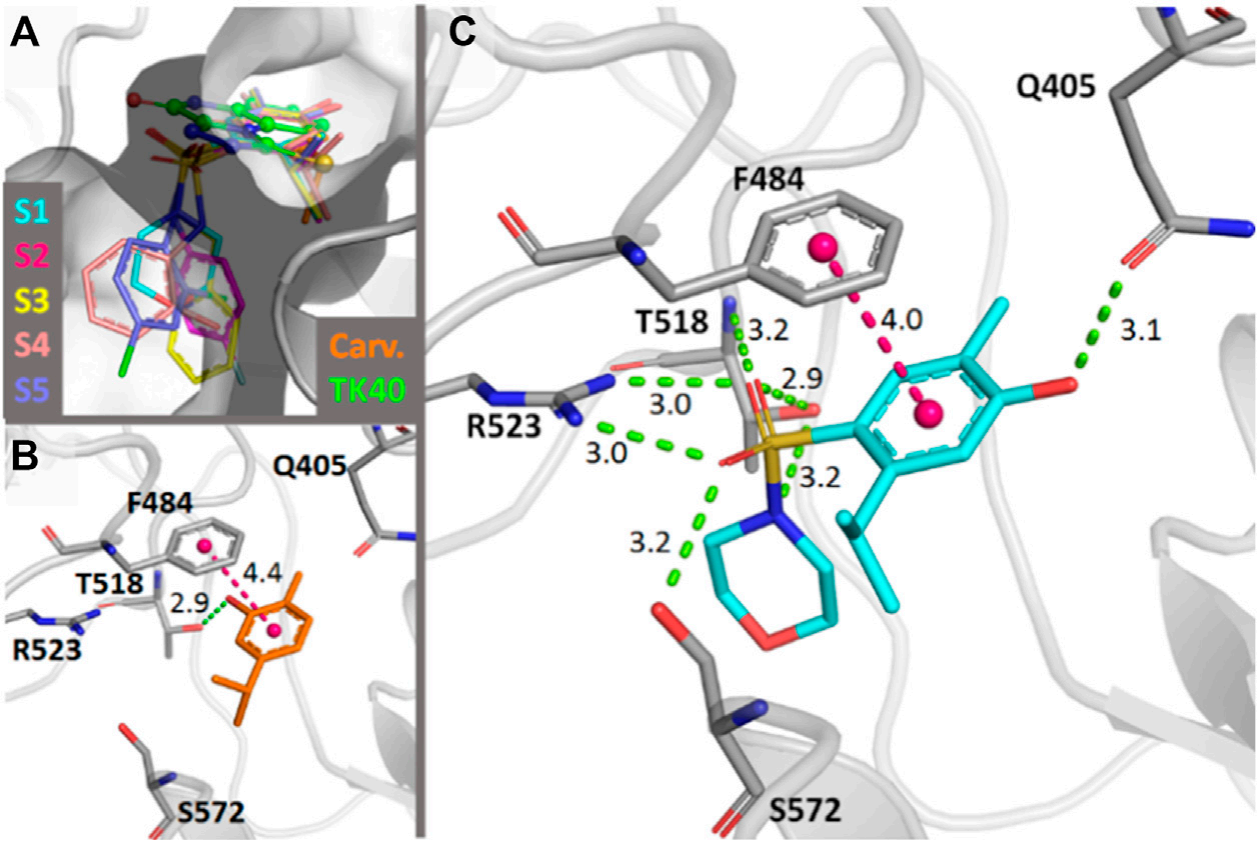
**(A)** Docking-predicted binding modes in the *rattus norvegicus* NMDA-glycine binding site (PDB ID 4KFQ). The carbon atoms of each molecule are represented in a different color. The carbon atoms of the crystallographic antagonist TK40 are shown in green. **(B)** Main interactions established by carvacrol in the predicted binding mode. **(C)** Main interactions found by **S1** in the predicted binding mode. Hydrogen bonds are represented in green and π-interactions in magenta. Distances are in Å.

For the predicted binding modes of **S1–S5**, the main interactions involving the common scaffold are hydrogen bonds with Gln405, Arg523, Thr518 and Ser572 and a displaced π-stacking interaction with Phe484. Of these, the interactions with Arg523, Thr518, and Phe484 are also observed for the crystallographic antagonist TK40 (Ravn et al., 2013). The main interactions observed for carvacrol are only hydrogen bonds with Pro516 and Thr518 and the displaced π-stacking interaction with Phe484 (Figure 7). For the different *R* groups, mainly van der Waals interactions were established. Only for the *R* groups of **S3** and **S5**, -CH···π interactions with Leu538 and Ser572, respectively, were observed. Among all five molecules, **S4** established the lowest number of contacts. The scores of each analyzed pose are presented in Supplementary Table S2.

**FIGURE 8:**
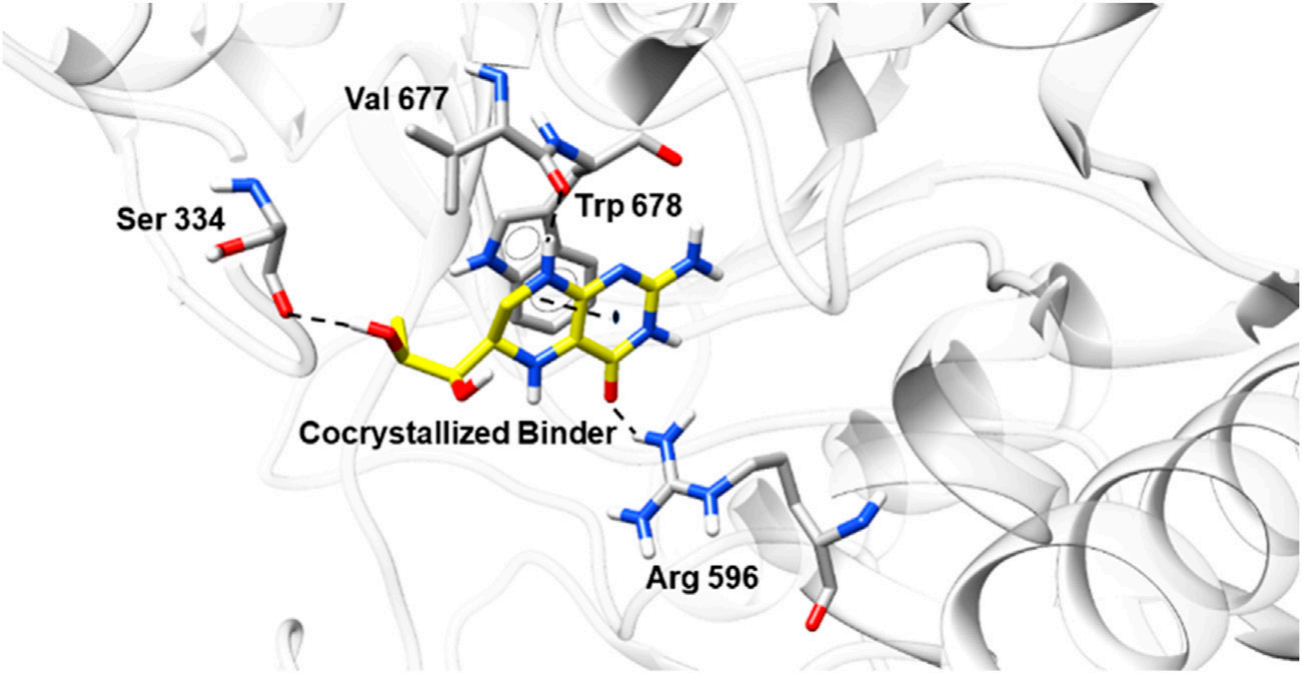
Conformation of the crystallographic ligand in the binding site of NO synthase (PDB ID 6NGJ) after the redocking studies.

The three levels of perception of pain—the cerebral (Dickenson, 1995), spinal and peripheral (Gordh et al., 1995)—appear to be affected by NO. This compound is an essential regulator of various immune and inflammatory functions (Moncada et al., 1991). In this work, we investigated, besides the NO scavenging activity, the possible intermolecular interactions between the sulfonamides and NO synthase. First, to validate the molecular docking protocol, redocking analysis (Figure 8) of 5,6,7,8-tetrahydrobiopterin (the crystallographic ligand, PDB ID 6NGJ) (Do et al., 2019) was carried out with GOLD. The ligand occupied the same interaction site in molecular docking when compared to the crystallographic structure, with emphasis on hydrogen bond interactions with Ser334, Val677, and Arg 596 and a π interaction with Trp678.

**FIGURE 9:**
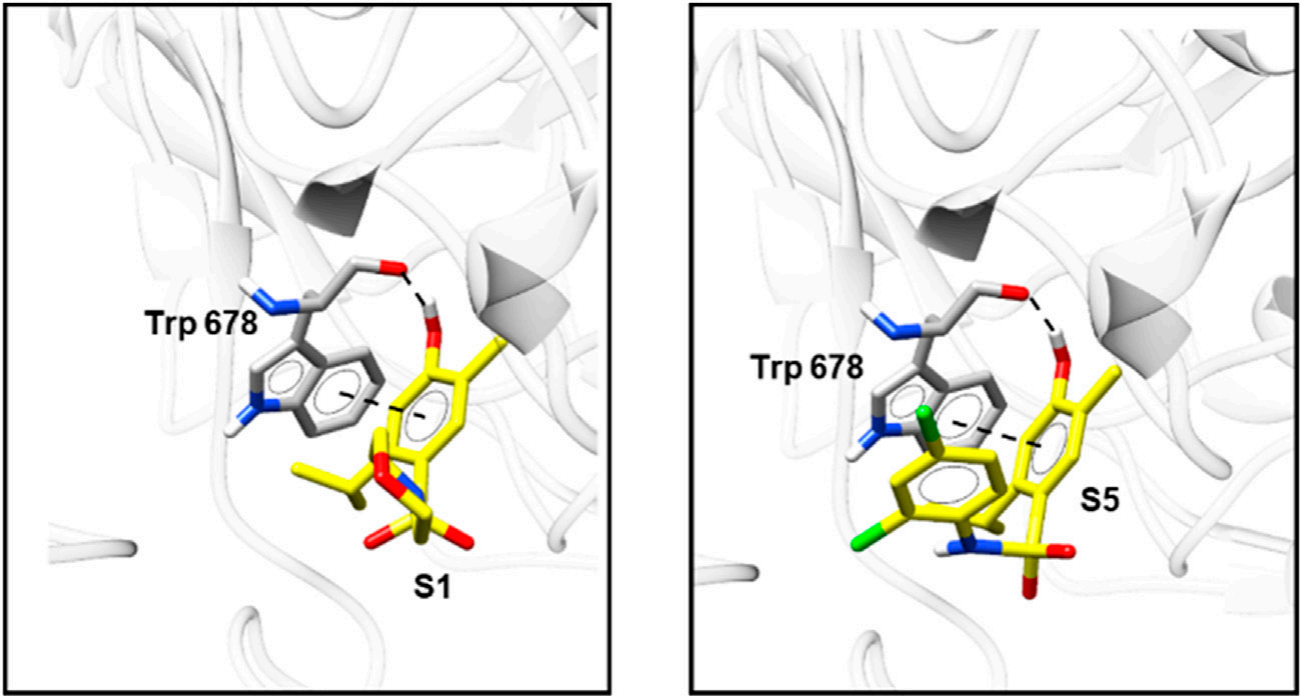
Top-scoring docking poses for **S1** and **S5** in the binding site of NO synthase (PDB ID 6NGJ).

The molecular docking results agree with the results obtained in the *in vitro* (NO scavenging activity) and *in vivo* tests. All compounds showed an inhibitory profile against NO synthase, except **S4**, which was not effective in all performed assays. The two most active compounds, **S1** and **S5** presented lower ID_50_ values and higher values for the scoring function, which demonstrate the high correlation between the *in vivo* and *in silico* results. The higher activity of these compounds was probably due to π stacking interactions and a hydrogen bond between compounds **S1** and **S5** and Trp 678 (Figure 9), which were also observed for the co-crystallized ligand, but was not found for the other sulfonamides. The scores of each analyzed pose are presented in Supplementary Table S3.

### Molecular Properties

Physicochemical and topological parameters of compounds **S1–S5** were estimated to evaluate their pharmacokinetics profile. The octanol–water partition coefficient (miLogP), topological polar surface area (TPSA), molecular weight (MW), number of atoms, hydrogen-bond acceptors (HBA) and hydrogen-bond donors (HBD), number of rotatable bonds (NRB), Lipinski RO5 violations, and molecular volume are presented in Table 2. The *silico*-derived descriptor values were compared with the solubility and permeability filters for drug candidates reported by Lipinski (Barret, 2018), Oprea and Veber (Veber et al., 2002).

**TABLE 2:** Molecular properties of sulfonamides **S1–S5**.

Property	S1	S2	S3	S4	S5
miLogP	2.43	4.20	2.57	3.77	5.32
TPSA (Å^2^)	66.84	66.40	79.29	86.62	66.40
Natoms	20	22	22	22	23
MW	299.39	323.39	320.41	321.40	374.29
HBA	5	4	5	5	4
HBD	1	2	2	3	2
nviolations	0	0	0	0	1
NRB	3	4	5	4	4
Molecular volume (Å^3^)	268.14	278.75	286.46	281.83	300.89

**FIGURE 10:**
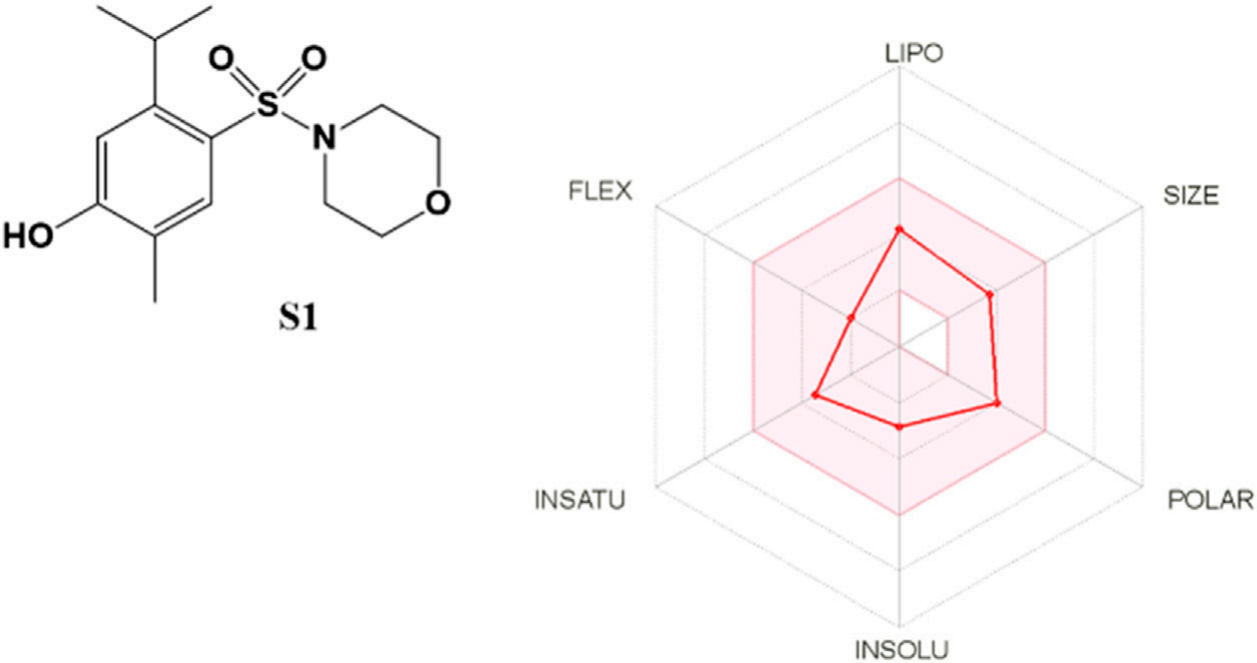
The Bioavailability Radar for **S1**. The figure was generated online using SwissADME. Compound **S1** combines good hydrophobicity and solubility, which is vital for membrane transport and permeability. Also, it does not violate any of the filters proposed by Lipinski, Ghose, Veber, Egan, and Muegge (Figure 11).

**FIGURE 11:**
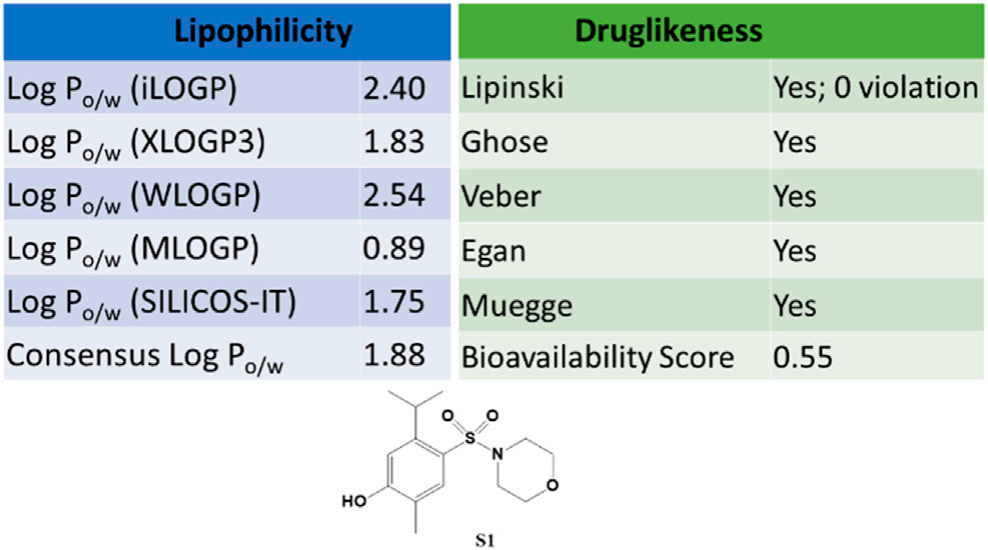
Lipophilicity and drug likeness for **S1**. These parameters were generated online using SwissADME.

**FIGURE 12:**
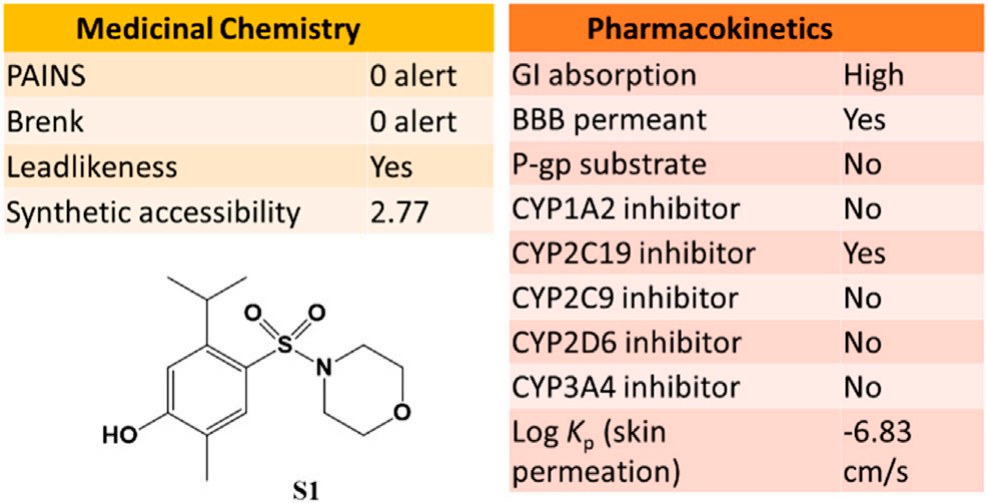
Medicinal Chemistry and pharmacokinetics for **S1**. These parameters were generated online using SwissADME.

The SwissADME web tool used to calculate the parameters is available at http://www.swissadme.ch and allows straightforward submission and analysis. It allows different input methods, multi-molecule computation, and offers the possibility to view and save results for each molecule, in addition to an interactive and intuitive visualization tool. To study the ADME parameters of the most active sulfonamide in the *in vitro* and phenotypic tests (**S1**), the Bioavailability Radar (Figure 10), lipophilicity, drug likeness (Figure 11), medicinal chemistry and pharmacokinetics (Figure 12) parameters were analyzed.

The Bioavailability Radar (Figure 10) provides a graphical output for the drug-likeness of a compound. The central shaded surface is the optimal domain for lipophilicity (XLOGP3 from −0.7 to +5.0), size (MW from 150 to 500 g/mol), polarity (TPSA from 20 to 130 Å^2^), aqueous solubility (logS ≤ 6), saturation (fraction of sp3 carbons ≥ 0.25), and flexibility (rotatable bonds ≤ 9). Compound **S1** falls within the optimal range for all parameters.

In addition, **S1** has a good medicinal chemistry and synthetic accessibility profile, which is very important in obtaining a drug that can be commercially distributed at a more affordable price. Moreover, **S1** has high gastrointestinal absorption (GI) and blood-brain barrier permeability (Figure 12).

## Discussion

### Antioxidant Activity

The evaluation of the antioxidant activity of a series of compounds should be performed in more than one experiment, allowing for the reliability of the results (Sens et al., 2018). Diverse *in vitro* antioxidant assays have been published. Herein, the antioxidant ability of derivatives **S1–S5** was determined in two *in vitro* tests. Subsequently, the results of these tests were correlated with the findings from the HOMO and LUMO studies.

Compounds **S1**, **S3**, and **S5** were more active than ascorbic acid (AA), which was used as the reference compound. Compound **S5** showed the highest activity, and **S4** demonstrated to be the least active. A linear correlation was found between both experimental results (EC_50_^H2O2^ = 1.085EC_50_^NO^ + 0.2250; *r* ^2^ = 0.99).

NO plays a critical part in the control of multiple physiological responses. Also, the NO cascade is associated with many conditions, including Alzheimer’s disease (Di Meo et al., 2016). H_2_O_2_ readily decomposes into water and oxygen, resulting in the production of hydroxyl radicals (OH•), lipid peroxidation and DNA injury, which makes it a target for research of new compounds with antioxidant properties (Phaniendra et al., 2015).

Extensive research has revealed that NO plays an essential role in several biological processes, such as neurotransmission, immune defense, and regulation of cell death (Snider and McMahon, 1998). The early 20th century witnessed the discovery of the role played by NO in nociception in both the central and peripheral levels (Zhuo and Gebhart, 1997). One of the physiological functions of NO was initially found in the vasculature; it was shown that the role of endothelium-derived relaxation factor (EDRF) could be quantitatively explained by the formation of NO by endothelial cells (Moncada and Higgs, 2006).

Treatment of pain with NO donors began with the use of nitroglycerin (NTG), which figures among the oldest treatments for ischemic heart disease (Boden et al., 2015). Discovered in 1847, NTG was used for the therapy of pain in angina pectoris for 100 years. However, its mechanism of action was not revealed until EDRF was identified as NO (Marsh and Marsh, 2000). Independently, NO was found to be an endogenous activator of soluble guanylate cyclase, resulting in the formation of cyclic GMP (cGMP), which acts as a second messenger in many cells, including the sensory neurons (Pereira et al., 2011).

NO is a highly reactive chemical messenger diffusible through the cytoplasmic membranes that is critical for the control of neuronal transmission, inflammation, cytotoxicity, and neural plasticity (Pacher et al., 2007). NO modulates the excitability of spinal sensory neurons and contributes to pain in different ways. The control of NO biosynthesis is regulated by NO synthase (NOS) enzymes. Three NO synthase isoforms (NOS; EC 1.14.13.39) catalyze the production of NO (Förstermann and Sessa, 2012). They use O_2_ and L-arginine as substrates and flavin mononucleotide (FMN), flavin adenine dinucleotide (FAD), reduced nicotinamide-adenine-dinucleotide phosphate (NADPH), and tetrahydrobiopterin (BH4) as cofactors (Förstermann and Sessa, 2012). In this work, molecular docking was used to investigate NOS inhibition by the carvacrol derivatives.

### Quantum Studies

The HOMO profile showed a variation of the charge density among the carvacrol derivatives. The HOMO and LUMO energies of compound **S1** is −7.40 and −0.62 eV, respectively. The electronic density is concentrated in the phenol group for HOMO and LUMO. Compound **S2**, however, differs regarding the position of the charge density for these orbitals. In HOMO (−6.86 eV), the orbitals are concentrated on the fluoro-phenyl group. This is because fluorine tends to attract electron density (electronegative atom). In LUMO (−0.75 eV), the electronic density tends to be favorable in the phenol group. The band gap in this compound is −6.11 eV. Compound **S3** has HOMO and LUMO energies of −7.35 and −0.86 eV, respectively. The electronic density of HOMO tends to be located at the phenol. In LUMO, however, the electronic density concentrates in the region of the pyridinic group. In compound **S4**, the HOMO charge density surrounds the phenolic substituent (−6.83 eV). In LUMO, however, the charge density concentrates in the carvacrol fragment (−0.59 eV). Differently from the other compounds, the charge distribution in **S5** distributes throughout the structure in HOMO (−7.38 eV) and LUMO (−1.01 eV). In HOMO, the positive density concentrates on the sulfonamide group and *p*-fluorine atom. In LUMO, however, the same region is predominantly negative throughout the structure. In ascorbic acid, the dihydroxyfuran has the HOMO electron density (−6.91 eV) close to the hydroxyl groups in the resonant region. In LUMO (−0.49 eV), the signal of electronic density changes and concentrates close to the oxygen atom of the furan group.

### Antinociceptive Activity

Injection of glutamate (i.pl.) in the mouse paw causes significant paw edema and nociception (Beirith et al., 2002; Meotti et al., 2010). Figure 4 shows that systemic administration of carvacrol, **S1**, **S2**, **S3**, and **S5** significantly inhibits nociception induced by injection of 20 µmol/paw glutamate, suggesting that these compounds have an important therapeutic effect for the treatment of acute pain of inflammatory origin, probably due to a decrease in peripheral glutamatergic signaling. Treatment with the compounds significantly reduced pain behavior induced by glutamate (i.pl.), characterized by spontaneous licking/biting of the injected hind paw. Carvacrol was able to reduce nociceptive behavior by 19 ± 6 and 44 ± 8% at 0.0003 and 0.003 mg/kg, respectively. Moreover, the sulfonamides derived from carvacrol showed the following percent inhibitions: 16 ± 5%, 62 ± 5%, and 50 ± 7% for **S1** at 0.0003, 0.003, and 0.03 mg/kg, respectively; 26 ± 5% and 34 ± 6% for **S2** at 0.003 and 0.03 mg/kg, respectively; 25 ± 9% and 22 ± 5% for **S3** at 0.003 and 0.03 mg/kg, respectively; 39 ± 8% and 27 ± 13% for **S5** at 0.003 and 0.03 mg/kg, respectively.

The calculated mean ID_50_ value for sulfonamides derived from carvacrol was 0.002 (0.001–0.002) mg/kg for **S1**, 0.442 (0.063–0.387) mg/kg. Thus, the results of the present study demonstrate that carvacrol and **S1**, **S2**, **S3**, and **S5** reduce nociception induced by glutamate (i.pl.), suggesting that inhibition of the stimulatory mechanism via peripheral glutamatergic neurotransmission may contribute, at least in part, to the antinociceptive effect of these compounds. In addition, we would like to highlight that carvacrol and compounds **S1**, **S2**, **S3**, and **S5** may be interesting lead compounds for acute pain, especially **S1** (0.003 mg/kg) since it presented the highest efficacy among the analyzed compounds.

Importantly, the compounds derived from carvacrol, selected to carry out the *in vivo* experiments, were chosen from the results presented in the molecular docking, quantum studies, and the *in vitro* antioxidant activity. Our results corroborate previous results (Arigesavan and Sudhandiran, 2015) which also found antioxidant and anti-inflammatory effects after treatment with carvacrol, using a carcinogenicity model in the colon of rats. Moreover, previous studies demonstrated that carvacrol attenuates mechanical hypernociception induced by carrageenan (Guimarães et al., 2012) and the acute pain acetic acid-induced abdominal constriction and formalin (Cavalcante Melo et al., 2012). Also, it was shown (Barnwal et al., 2018) that carvacrol increased the activities of antioxidant enzymes and downregulated expression by reducing the inflammation marker in positively dyed cells (iNOS, NF-κB, and COX-2) in a pulmonary toxicity model. These data from the literature reinforce the antinociceptive, anti-inflammatory, and antioxidant potential of carvacrol observed in our study.

Findings from the literature (Pacher et al., 2007; Förstermann and Sessa, 2012) indicate that superoxide (SO, O^(2)•(−)^) and peroxynitrite (PN, ONOO^(−)^, the product of its reaction) are essential for the emergence of pain caused by different etiologies. These findings reinforce the concept that ROS play an essential part in NMDA activation, which is a critical ionotropic glutamatergic receptor, which contributes to central and peripheral pain. Therefore, this study supports previous results (Wang et al., 2004) that stated that superoxide mediates hyperalgesia (increased sensitivity to painful stimulation) through M40403, a manganese(II) complex with a bis(cyclo-hexylpyridine-substituted) macrocyclic ligand, which is a superoxide dismutase mimetic. These findings disclosed the central role played by superoxide in the peripheral signaling of nociception. In addition, it was shown that the M40403 antihyperalgesic activity could not be reverted by naloxone, which excludes the participation of opioid signaling cascades. Moreover, so far, few studies have investigated the effect of carvacrol on neurotransmitter modulation. The studies by Zotti et al. (2013) demonstrated that carvacrol, when ingested regularly in low concentrations, influences brain activity by increasing the levels of neurotransmitters such as serotonin and dopamine, which can determine feelings of well-being and reinforcing positive effects. Thus, our interest in investigating the glutamatergic system has arisen, considering that glutamate is a major mediator in the CNS, mediating excitatory neurotransmission in mammals, including in sensory neurons that convey pain, being strongly involved in the stimulation of peripheral and central pain. Therefore, our findings are unprecedented and relevant as they demonstrate the inhibitory capacity of carvacrol on the peripheral glutamatergic pathway.

It was shown (Kuo et al., 2017) that carvacrol mitigated injury in tissues and inflammation derived from periodontitis induced by ligation. Besides that, carvacrol proved to attenuate inflammatory response induced by carrageenan, decreasing mouse paw edema (Guimarães et al., 2012). These data from the literature support the anti-inflammatory, antinociceptive and antiedematogenic effects of carvacrol observed in our study. Importantly, paw edema and pain induced by glutamate are essentially associated with non-NMDA ionotropic glutamate receptors and NO production, a vasodilator, and an important neurotransmitter (Beirith et al., 2002). When in excess, it may be involved in the production of oxidative lesions in proteins. These findings reinforce the importance of studying glutamate-induced paw edema and nociception, as well as the beneficial effects of carvacrol and its derivatives found in this study.

Our results agree with literature data which demonstrated that carvacrol had no effect on the spontaneous locomotion in mice (Cavalcante Melo et al., 2012; Guimarães et al., 2012). However, these studies used a curve of carvacrol doses ranging from 25 to 100 mg/kg in the open-field test and we are the first group to test a much lower dose curve for carvacrol (0.0003, 0.003, and 0.03 mg/kg) in pain, edema, and spontaneous locomotion. In addition, Guimarães et al. (2012) demonstrated that carvacrol at a dose of 100 mg/kg reduced the animals’ ambulation in the open-field test, 30 min after intraperitoneal administration, showing that this dose is not safe as it causes nonspecific effects on locomotor activity and should be excluded in future pain studies. It is already well described that some drugs can cause motor slowness (bradykinesia) or even act as a muscle relaxant, causing non-specific changes in the locomotor activity of animals (Cartmell et al., 1991). In addition, drugs like benzodiazepines and other anxiolytics decrease the exploratory behavior of animals (Hazim et al., 2014). In this regard, it was demonstrated (Coderre and van Empel, 1994) that many glutamate antagonists, primarily via ionotropic NMDA receptor, such as the receptor channel block MK-801, produce significant antinociceptive effects, but decrease exploratory behavior of animals. In contrast, our results demonstrate that the intragastric treatment with the tested compounds can induce a significant antinociceptive effect via inhibition of peripheral glutamate, without causing any detectable motor dysfunction. Thus, carvacrol and its derivatives **S1**, **S2**, **S3**, and **S4** at doses up to 0.03 mg/kg have an attractive analgesic potential to treat acute pain without causing CNS sedation.

### Molecular Docking

In general, no significant binding modes were obtained concerning poses matching the available structural criteria of known antagonists (Ramírez and Caballero, 2018). Only the docking simulations in the NMDA-GluN_1_ glycine binding site (LBD-GluN_1_) excelled, which agrees with previous observations for selective ligands of this site, such as HA-966, “which barely interacts with other ionotropic glutamate receptors” (Planells-Cases et al., 2005).

Considering the docking results and the non-ataxic effects of the compounds at the administered doses, the compounds are likely to be partial agonists, instead of agonists of the NMDA-GluN1 glycine binding site, such as rapastinel (Wood et al., 2008) (GLYX-13 or BV-102), (+)-HA-966 (Millan and Seguin, 1993) and the recently reported 1-amino-1-cyclobutanecarboxylic acid (Fung et al., 2019).

### Molecular Properties

The Lipinski RO5 applies to compounds that are active after oral administration. The RO5 includes four physicochemical property ranges (logP ≤ 5, MW ≤ 500, HBD ≤ 5 and HBA ≤ 10) that are present in 90% of the drugs that are active after oral administration and have reached phase II clinical development (Barret, 2018). The sulfonamides investigated in this work are within the RO5 desirable range, except for the miLogP of sulfonamide **S5** (miLogP = 5.32), which is slightly higher than expected.

TPSA correlates with a compound’s ability to permeate biological membranes through passive transport. Medicinal chemists use TPSA as an important parameter to optimize drug permeation through membranes. Molecules having TPSA values higher than 140 Å^2^ are likely to permeate poorly into cell membranes (Pajouhesh and Lenz, 2005). For molecules that are required to act in the CNS, penetration into the blood-brain barrier is needed, which requires a TPSA lower than 90Å^2^ (Hitchcock and Pennington, 2006). All investigated sulfonamides are in accordance with these parameters. A molecule that has a higher number of rotatable bonds becomes more flexible and have a good binding affinity with the binding pocket. For a potential drug candidate, Veber proposed that NRB should be ≤10. All investigated sulfonamides are following this parameter.

The molecular volume assesses the transport properties of molecules such as blood-brain barrier penetration. The calculated values for this property are in line with the values expected for drug candidates.

During the discovery of novel drugs, molecules with useful therapeutic properties and low levels of toxicity are highly desirable. In this process, knowledge of the absorption, distribution, metabolism, and excretion profiles (ADME) is essential. It is well-known that the early evaluation of ADME during the drug discovery process reduces the attrition rates during clinical development.

## Conclusion

In this study, we report the SAR for a series of carvacrol-derived sulfonamides. The antioxidant and antinociceptive activities of compounds **S1–S5** were investigated using *in vitro* and *in vivo* assays. All the sulfonamides showed antioxidant activity in the *in vitro* tests comparable to that of the control compound (ascorbic acid). The results gathered in the *in vitro* antioxidant tests were linearly compared to the binding energies of the HOMO frontier orbital (*r* ^2^ = 0.87 and 0.88) calculated by DFT. The results of this study demonstrate that carvacrol and its derivatives **S1**, **S2**, **S3**, and **S5** were able to reduce nociception induced by glutamate (i.pl.). Moreover, these findings show that the intragastric treatment with the tested compounds can induce a significant antinociceptive effect via inhibition of glutamatergic peripheral system without causing any detectable motor dysfunction, and not affecting the locomotor activity of mice. Thus, carvacrol and compounds **S1**, **S2**, **S3**, and **S5** at doses up to 0.03 mg/kg have an attractive analgesic potential to treat acute pain with no CNS sedation. Docking simulations highlighted the interactions between the compounds and the NMDA-GluN_1_ glycine binding site, which suggested that these molecules act as selective partial agonists. Besides, compounds **S1–S5** exhibit physicochemical parameters and pharmacokinetics compatible with drug candidates. Overall, sulfonamides **S1–S5** are suitable starting points for further molecular optimization.

## Data Availability

The original contributions presented in the study are included in the article/Supplementary Material, further inquiries can be directed to the corresponding authors.
