# Supplementary material for: Antioxidant Activity, Molecular Docking, Quantum Studies and In Vivo Antinociceptive Activity of Sulfonamides Derived From Carvacrol
Source: Front Pharmacol. 2021 Nov 23;12:788850. doi: 10.3389/fphar.2021.788850 (PMC8650121; doi:10.3389/fphar.2021.788850)
Supplement: Supplementary file 1 [file DataSheet1.PDF]

Antioxidant activity, molecular docking, quantum studies and *in vivo* antinociceptive activity of sulfonamides derived from carvacrol

Aldo S. de Oliveira[1,6*], Luana C. Llanes[2], Ricardo J. Nunes[3], Catharina Nucci-Martins[4,5], Anacleto S. de Souza[6], David L. Palomino-Salcedo[6], María J. Dávila-Rodríguez[7], Leonardo L. G. Ferreira[6*], Adair R. S. Santos[4] and Adriano D. Andricopulo[6]

[1]Department of Exact Sciences and Education, Federal University of Santa Catarina-UFSC, Blumenau-SC, Brazil. [2]Department of Chemistry and Biochemistry, University of California, Santa Barbara, California 93106, USA. [3]Departament of Chemistry, Federal University of Santa Catarina-UFSC, Florianópolis-SC. Brazil. [4]Department of Physiological Sciences, Center of Biological Sciences, Federal University of Santa Catarina-UFSC, Florianópolis-SC. Brazil. [5]Department of Structural and Functional Biology, Institute of Biology, University of Campinas-UNICAMP, Campinas-SP. Brazil. [6]Laboratory of Medicinal and Computational Chemistry, Institute of Physics of São Carlos, University of São Paulo-USP, São Carlos-SP, Brazil. [7]Department of Chemistry, Federal University of São Carlos-UFSCar, São Carlos-SP, Brazil.

***Correspondence**

Aldo S. de Oliveira: aldo.sena@ufsc.br

Leonardo L. G. Ferreira: leonardo@ifsc.usp.br

Table S1. Summary of the glutamate receptor structures of *Rattus norvegicus* bound to antagonists used in the molecular docking simulations. In each case, the explored domain is indicated.

**Receptor Type**		**Domain Explored**	**PDB ID**	**Res. (Å)**
iGluR	NMDA	ATD-GluN$_1$/GluN$_2$B interface	6E7R	2.1
		LBD-GluN$_1$	4KFQ	2.2
		LBD-GluN$_2$A	5U8C	1.598
		LBD-GluN$_1$/GluN$_2$A interface	5I58	2.52
	AMPA	LBD-GluA$_2$	3H03	1.9
		LBD-GluA$_3$	4F1Y	1.79
	Kainate	LBD-GluK$_1$	4YMB	1.93
		LBD-GluK$_2$	5CMK	1.801
		LBD-GluK$_3$	6F28	2.4
mGluR	Group I	LBD-mGluR$_1$	1ISS	3.3

iGluR = Ionic glutamate receptor. mGluR = metabotropic glutamate receptor. NMDA = *N*-metthyl-D-aspartate receptor. AMPA = α-amino-3-hydroxy-5-methyl-4-isoxazole propionic acid receptor. Kainate = Kainic acid receptor. ATD = Amino terminal domain. LBD = Ligand binding domain.

Table S2. Scores of the predicted binding modes after docking in the *rat*NMDA-glycine binding site (PDB ID 4KFQ).

Compound	**ID$_{50}$ (mg/kg)**	**ChemPLP Score**
S1	0.002	77.4
S2	--	74.2
S3	--	86.0
S4	--	66.4
S5	--	83.8
Carvacrol	--	50.8
TK40*	--	65.9

*Crystallographic antagonist.

Table S3. Best CHEMPLP Fitness and ID_{50} values

Compound	6NGJ	Antinociceptive activity ID_{50} (mg/Kg)
**S1**	50.459	0.002 (0.001-0.002)
**S2**	41.493	ND
**S3**	49.921	ND
**S4**	39.788	ND
**S5**	51.805	ND
**Carvacrol**	40.475	ND
**Co-crystallized ligand**	46.965	ND

ND: not determined.
